# ADL-Focused Occupation-Based Neurobehavioral Evaluation Software: Addition of a Rasch-Based Stroke Subscale to Measure Outcomes

**DOI:** 10.3390/brainsci15090904

**Published:** 2025-08-23

**Authors:** Guðrún Árnadóttir, Laufey Halla Atladóttir, Garðar Ingvarsson, Helgi Sigtryggsson, Bjarni Ármann Atlason

**Affiliations:** 1 Landspitali, 105 Reykjavik, Iceland; 2 Faculty of Medicine, University of Iceland, 102 Reykjavik, Iceland; 3 Independent Researcher, 107 Reykjavik, Iceland

**Keywords:** ADL evaluation, CVA, instrument development, measurement, occupational therapy, outcome studies, Rasch analysis, rehabilitation, software development, stroke

## Abstract

**Background:** Measurements are necessary in rehabilitation for evaluating service effectiveness. The ADL-focused Occupation-based Neurobehavioral Evaluation (A-ONE) is used for evaluating ADL performance and the impact of neurobehavioral impairments on the performance. Recently, Rasch-based software was constructed for the A-ONE ADL and neurobehavioral scales. It converts ordinal rating scale scores into measures, estimates missing data values and calculates the statistical significance of changes. **Objectives:** To expand the A-ONE software by developing a cerebrovascular accident (CVA) neurobehavioral subscale. Additionally, to pilot-test whether the ADL and CVA scales of the software can detect statistically significant improvements. **Method:** Rasch analysis was used for evaluating the item fit, PCA, person separation and reliability to establish the internal validity of the CVA subscale (*n* = 222). The external validity (*n* = 22) was obtained by comparing A-ONE software measures to Winsteps measures. Subsequently 21 pre–post-intervention comparisons were made of stroke patients using both the ADL and CVA scales. **Results:** All set criteria for internal and external validity were met. By using the software clinically after incorporating the CVA subscale, statistically significant changes were detected in 90.5% of comparisons using the ADL scale and 36.4% using the CVA scale. The intervention program used was determined to consist of 66.4% occupation-based activities. **Conclusions:** This study is the first to deliver a clinically deployable Rasch-based CVA subscale integrated into routine occupational therapy software. The A-ONE software offers considerable time saving for therapists and the potential to detect significant differences in performance and impairment impact. It contributes to the removal of clinical obstacles toward the use of the instrument as an outcome measure and encourages the use of measures in rehabilitation.

## 1. Introduction

Stroke, also termed cerebral vascular accident (CVA), is reported to be a leading cause of long-term disability [1,2]. The global burden of stroke is increasing, with 12 million new strokes reported every year and 94 million people living with the effects of stroke. Approximately 53% of strokes occur in people under 70, and one out of every four people are expected to have a stroke in their lifetime [3]. Stroke is reported to reduce mobility in more than half of stroke survivors over 64 years old [1]. Furthermore, difficulties in performing activities of daily living (ADL) are declared to be more severe when the stroke results in cognitive impairments, and half of stroke survivors are reported to suffer from some cognitive impairment in the first couple of weeks following their first stroke [2]. Occupational therapy referrals for stroke patients are usually made when the resulting impairments are suspected to affect task performance [4]. Stroke frequently leaves survivors with some impairments that limit their performance in activities of daily living (ADL), such as dressing, grooming, transfers or feeding. The impact of neurological impairments on the ADL task performance of persons with stroke can be evaluated by the ADL-focused Occupation-based Neurobehavioral Evaluation (A-ONE). Neurobehavior is defined as any behavior that reflects on neurological function [5,6]. It includes the different types of pertinent neurological body functions (i.e., cognitive, perceptual, motor) necessary for performing different tasks. It further refers to the processing and integration of both internal and external stimuli as well as motor, speech, thought and emotional responses by different mechanisms of the central nervous system (CNS). It involves feedback and requires a complex interaction of processing and response. All tasks (e.g., ADL tasks) provide sensory stimuli and elicit some form of response. Neurobehavioral theory can thus be considered to link brain processes to task performance. Neurobehavioral deficits that manifest in task performance errors can result from dysfunctional neurological processing affecting body functions (e.g., praxis, attention or visuospatial skills) [5,6,7]. From a pathophysiological perspective, it is not possible to observe the function of the nervous system directly, but errors in task performance can reflect its function by the influence they have on the performance of daily activities such as combing hair. In the A-ONE, such errors are translated by the application of clinical reasoning, as described further in the Methods section. The obtained information can contribute to goal setting and the selection of intervention approaches.

The A-ONE, first published in 1990 [5,8,9,10], was originally termed the Árnadóttir OT-ADL Neurobehavioral Evaluation [4,5,8,9,10,11]. The A-ONE has been used by occupational therapists in numerous countries [8]. A recent survey [2] revealed, for example, that the A-ONE is the second-most commonly used standardized instrument by occupational therapists in Danish hospitals for poststroke cognitive impairments, both in terms of ADL evaluations and cognitive evaluations. Its two ordinal scales, an ADL scale and a Neurobehavioral Impact (NBI) scale, are used to evaluate, based on the same observations, both the task performance of daily activities in the natural context as well as the type of impairments impacting and limiting the performance. The purpose of developing the A-ONE was to construct an occupation-based ADL instrument by operationalizing neurobehavioral concepts to aid therapists in clinical reasoning and decision making that could be applied to gather descriptive information of benefit for the selection of intervention strategies [5,8]. Later, occupational therapists additionally wanted to utilize the evaluation to describe changes in performance and outcomes.

As measurements are necessary in rehabilitation for evaluating service effectiveness [12,13], several studies were undertaken using Rasch analyses to examine the possibility of converting the ordinal descriptive type of A-ONE-scale scores into valid and reliable measures [6,14,15]. These studies included the construction of conversion tables for the renewed scales. They also allowed for the production of key forms, as suggested by Linacre [16], that enable clinicians to use measurement information directly in the clinic as opposed to having such information stored away in databases. The key forms can be used to estimate measures despite missing or outlying scores [16,17]. Unfortunately, therapists often seem to not only be reluctant to use standardized assessments in their work [2,18,19,20,21] but also conversion tables for converting raw scores into measures. Their explanations for non-use include burden of administration, lack of time and difficulties with interpretation due to statistical limitations [18,20]. The same reasons seem to also apply to not using key forms. Rasch-based key forms have reportedly been used in rehabilitation to predict missing data [16,17,18], interpret scores [19], set goals [21], monitor progress [18] and design rehabilitation sessions and interventions [19,22,23]. Reports indicate that some clinicians seem to be unaware of the existence of key forms, while others lack an understanding of the knowledge regarding their use, thus requiring extra time [18,20]. Therefore, a Rasch-based A-ONE software was recently constructed for both the A-ONE ADL and Neurobehavioral scales [24].

The A-ONE, used to evaluate simultaneously task performance and neurobehavioral impairments that limit performance, has been claimed to be “the first occupation-based cognitive assessment” in the area of neurorehabilitation [9]. Not only can it be used to evaluate the impact of cognitive and perceptual impairments on ADL performance in a natural context, but it can also be used to evaluate the effect of motor dysfunction. The development of the A-ONE has taken place through several different cycles based on analyses offered by the Model of an Integrated Profession [6,8,25]. The dilemmas that arose in occupational therapy in the early 1980s led to the development of the historical cycle of the A-ONE. These dilemmas included separate tests being used for ADL and body functions, the common use of borrowed tests from outside the discipline of occupational therapy, decontextualized test results that did not translate into occupational therapy and a lack of systematic evaluation of neurobehavioral errors observed in ADL performance [6,8]. The historical cycle of the A-ONE includes development of both the conceptual background of the evaluation and its ordinal scales constructed through Classical Test Theory (CTT) research studies [6,8].

The A-ONE is an occupation-centered instrument. Its conceptual framework originates from an occupational therapy perspective linking neurological knowledge with the core of occupational therapy. The two ordinal rating scales of the instrument, an ADL scale and a Neurobehavioral Impact scale are both occupation-based, as the analysis of observed ADL task performance of basic ADL is the evaluation method utilized for both scales. The ADL scale is additionally occupation-focused; that is, the scores are concentrated on the ADL task performance [26,27]. Scoring of the Neurobehavioral Impact scale, on the other hand, is focused on the impact of the neurological impairments that may limit the observed ADL performance [8]. The original 22-item Functional Independence (FI) scale of the A-ONE with five domains (dressing, grooming and hygiene, transfers and mobility, feeding, as well as communication) is used for evaluating the level of independence and type of assistance needed [5,6,14].

Administration of the A-ONE requires performance analysis of the observed tasks. Detected performance errors are documented. Subsequently, the detected errors are translated by the occupational therapist to operationally defined impairments through a clinical reasoning process that is practiced during the A-ONE training courses.

The number of available standardized occupational therapy instruments designed to evaluate the impact of cognitive and perceptual impairments in naturalistic contexts has increased in the past decades. Some of these include assessments aimed at evaluating a limited range of impairments such as the Catherine Bergego Scale (CBS) [28,29,30] and the Assessment of Awareness of Disability (AAD) [31,32]. Others are used to evaluate a group of executive and memory functions, most frequently through the performance of instrumental activities of daily living (IADL) [33,34]. However, none of these assessments seem to address global neurobehavioral functions, and thus, the A-ONE is reported to still be the only scale that evaluates a wide spectrum of neurobehaviors that can be observed to impact the quality or level of naturalistic ADL task performance [6,35].

Numerous studies have been carried out to investigate the psychometric properties of the A-ONE. In terms of the historical cycle of the A-ONE, CTT studies were used for the initial development of the evaluation. These studies include, for example, Inter-rater reliability, which reveal good to excellent results both for the FI scale [5,6,36,37,38,39] and the NB scale [5,6,36,37,38]. Concurrent validation studies, including for internal consistency [6,39], were also performed during this period.

The dilemmas that led to the development of the measurement cycle of the A-ONE included consistent pressure to use measures in rehabilitation to evaluate service effectiveness. However, most evaluations used by occupational therapists were based on ordinal scales that provided descriptive information, not measures. Thus, there were problems with the use of descriptive ordinal data in research; simultaneously, there was pressure to use outcome measures in rehabilitation [6,8]. The difference between the descriptive ordinal rating scale scores and equal interval values is that ordinal scales are based on the sequence of an attribute, such as the type of assistance, but not on numerical values based on equal intervals, which are the basis of interval scales. Higher ordinal ratings represent more of the attribute, but how much more is not known. Mathematical manipulation (i.e., adding up total scores) as if they were based on a number line of equal intervals or statistical manipulation is likely to produce misleading information [13]. Rasch analysis, a statistical method classified under Modern Test Theory (MTT) can be used to convert ordinal scores into a measurement unit called the log-odd probability unit [12]. It can be used both to develop scales [12,40] and to convert ordinal scales such as the Functional Independence Measure (FIM), developed through CTT research, into measures [16,41]. An increased number of scales developed using the MTT are now being used in the field of rehabilitation [12,42,43]. Studies applying MTT methods in the form of Rasch analyses to the A-ONE have confirmed the internal validity of both the 20-item five-step ADL rating scale [6,14,35] and different versions of dichotomized NB-scale items [6,15,24,44]. The results thus support that the ordinal scale items can be converted into interval scale values by use of specific Rasch-based conversion tables, and thereby that the A-ONE scales can be used correctly to measure change.

Considering the current status of measurement use and the documentation of performance evaluation in rehabilitation in a computerized world, it became obvious that there was a clinical need for an “easy-to-use” software program that provides measurement data for the A-ONE. The A-ONE software was developed for both the A-ONE ADL and neurobehavioral scales to bridge the gap between rating scale values and valid measures. The software allows for the documentation of rating scale scores and written comments based on the A-ONE observation, collapsing of rating scale categories based on the results of Rasch analyses, conversion of ordinal scores into Rasch-based measures, estimation of missing data values, generation of digital reports and calculation of the statistical significance of changes when comparing different measures [24]. The development of the A-ONE software occurred in several stages. The first stage was to develop software for the 20-item, five-category Rasch-based ADL scale based on the *Rasch Rating scale model* [24]. Subsequently, exploration of adding neurobehavioral scales to the software included numerous Rasch analyses of the neurobehavioral items [6] and comparison of those to earlier Rasch-based studies that have been described elsewhere [6,15].

Several problems have been detected in relation to many outcome studies in rehabilitation. Based on information from several published systematic reviews and clinicians, these problems include (1) missing information on the nature of the intervention provided [45,46,47,48] and (2) occupation-based instruments not being used for the evaluation [9,48]. Additionally, the evaluation is frequently based on ordinal rating scales, not interval measurement. These problems made us aware of the current situation and triggered the necessity of performing a study using an occupation-based evaluation with the measurement potential to measure outcomes of a clearly defined intervention program used in the rehabilitation of persons diagnosed with CVA.

The purpose of this paper is to explore the possibility of expanding the A-ONE software by developing a Rasch-based CVA neurobehavioral subscale. Additionally, to pilot-test whether both the ADL and CVA scales of the A-ONE software can detect a statistically significant improvement in persons diagnosed with CVA. The uniqueness of the proposed study is the integration of a Rasch-based subscale for stroke patients into clinically applicable occupational therapy software, which has the potential to provide a valuable methodological contribution to rehabilitation.

The following four research questions were posed to obtain the information needed to meet the objectives. The first two relate to the development of a Rasch-based CVA subscale for the A-ONE software. The remaining two questions refer to a pilot outcome study.

1. Can an internally valid CVA scale be constructed and added to the A-ONE software?
2. Does the newly developed A-ONE CVA software scale have external validity?
3. Can the ADL and CVA scales of the A-ONE software be used to statistically significantly measure outcomes of an intervention program for stroke patients?
4. Does the frequency of detecting statistically significant differences in the ADL and the CVA scales differ?

## 2. Materials and Methods

In order to construct the Neurobehavioral CVA subscale, two studies were undertaken. The objectives of the first one were to develop an internally valid and reliable Rasch-based scale. Subsequently, the information from this study was to be incorporated into the A-ONE software. The objective of the second study was to explore the external validity of the information provided by the software. After the development of the internally and externally valid CVA software scale, we proceeded with the third study, which was aimed at exploring whether the A-ONE software including both the ADL and CVA subscales could be used to statistically significantly measure the outcomes of an intervention program for stroke patients. As the content of intervention programs used in outcome studies are important for the replication of studies, we simultaneously included a fourth study aimed at describing the content of the intervention provided.

Ethical approval (36/2003) was granted on 24 July 2003 from the ethics committee of Landspítali hospital in Iceland for construction of the Rasch-based scales of the A-ONE from retrospective hospital records of CVA patients at the hospital’s rehabilitation unit. Another approval (S1442) was obtained later on 22 January 2024 from the same ethics committee for the retrospective collection of study outcomes data from stroke patients receiving occupational therapy services at the unit in 2024. Patient consent was waived due to the retrospective nature of the study, with only existing data included in the analysis.

### 2.1. Participants

The four studies all included different subject groups.

#### 2.1.1. Internal Validation of a CVA Subscale

According to Linacre [49], a sample size of 150 individuals is sufficient to construct a Rasch-based scale for most purposes (99% confidence interval for item difficulty calibrations to remain stable with an absolute value of 0.5 logit). Retrospective data from 222 individuals diagnosed with CVA were obtained for the scale construction and internal validity study of the CVA subscale from an earlier, bigger data sample of the A-ONE NB scales (mean age = 66.6 ± 13.9; range = 22–91; males 59.9%; L-CVA = 51.4%). For further information on age, gender and diagnostic groups of the sample, see Table 1.

#### 2.1.2. External Validation

A random sample including 10% (*n* = 22) of the internal validity group of 222 CVA individuals was subsequently obtained for use in the external validity study comparing the obtained A-ONE CVA software measures to the original A-ONE Winsteps measures (mean age = 69.7 ± 13.3; range 22–84; male = 68.2%; R-CVA = 59.1%).

#### 2.1.3. Intervention Program

Four occupational therapists from the rehabilitation unit at Landspítali participated in both the intervention study and the examination of the content of the intervention program provided to patients at Landspítali. Their graduation time from occupational therapy ranged from 8 to 39 years (mean = 24.5 ± 12.9), their working experience at the rehabilitation unit ranged from 3–37 years (mean = 19 ± 15.8), and the time from attending the five-day A-ONE training course ranged from 7–33 years (mean = 20.5 ± 11.1). The recorded workload of the therapists during the one week of gathering intervention data for the study consisted of 39 patients, including 19 stroke patients (mean age = 63.7 ± 9.1; males = 13).

#### 2.1.4. Outcomes Study

In the outcomes study, 19 participants provided 21 comparisons of test–retest information (age range = 42–76; mean = 60.6 ± 9.9; males = 61.9%). Diagnostic groups included patients with left CVA (LCVA) = 38.1%; right CVA (RCVA) = 23.8%, subcortical CVA = 33.3% and bilateral CVA = 4.8%. Hospital records of two of the patients allowed for two different comparisons.

### 2.2. Instrument

The A-ONE is used to evaluate ADL performance and the impact of neurobehavioral impairments, as noted earlier. The 20-item, five-category ADL rating scale is used for evaluating the level of independence and the type of assistance needed. Subsequently, interpretation, based on the therapist’s clinical reasoning, of the observed performance errors into operationally defined impairments impacting the performance is scored on the revised NBI scale. The NBI scale is composed of two subscales: the Neurobehavioral Specific Impairment subscale (NBSIS) and the Neurobehavioral Pervasive Impairment subscale (NBPIS). The NBSIS is composed of 37 five-category rating scale items related to the four ADL domains. These items include the following: *Motor apraxia*, *Ideational apraxia*, *Unilateral body neglect*, *Spatial relations*, *Unilateral spatial neglect*, *Organization and sequencing of activity steps*, *Perseveration*, *Motoric left body side*, *Motoric right body side* and *Topographical disorientation.* All but one of these impairment items are independently rated more than once in connection with performance in the different ADL domains. Persons are scored based on the need for assistance to overcome the occupational errors (NB impairments) during ADL task performance [4,5,6].

The clinical reasoning involved for filling in the NBSIS is based on critical performance cues or errors. The errors detected during dressing could, for example, be difficulties in placing the arms into the correct sleeves of a shirt. During the task analysis involved in the clinical reasoning process, several impairment hypotheses may be formed based on the observed cues. Interpretation of the cues might include impaired body functions such as *Unilateral body neglect*, *Unilateral spatial neglect*, *Spatial relations dysfunction*, *Ideational apraxia*, *Motor apraxia*, *Motoric* (i.e., paralysis), *Attention* deficit, lack of *Motivation* and impaired *Organization and sequencing* of activity steps. Subsequently, the therapist interprets these cues and forms hypotheses regarding the nature of the problems that interfere with the task performance, causing the errors. This is carried out by keeping in mind conceptual and operational definitions of the impairments from the A-ONE terminology, as well as the therapist’s neurological knowledge related to diagnostic patterns of impairments, neuroanatomy, function of the nervous system and information-processing models. If the conclusion from the clinical reasoning becomes that the dressing performance is limited by the impact of numerous errors that seem to be related to impaired *Organization and sequencing* of activity steps, *Ideational apraxia*, *Motor apraxia*, as well as impaired *Motor performance* on the right side, these items will be scored down on the NBI scale based on the level of the impact’s severity and the type of assistance needed [4,8].

The original 31 dichotomous NBPIS items are scored based on a detected error in at least one of the domains. Some of the pervasive items, however, require deficit-specific testing, such as the item of astereognosis. Other items such as insight require additional questions and comments to those used for task performance in ADL situations. The A-ONE manual includes conceptual and operational definitions for all items as well as detailed criteria for administering and scoring with the instrument [4,5,8].

### 2.3. Statistical Analyses

#### 2.3.1. Construction of an NBI CVA Software Subscale and Internal Validation

The Rasch scoring models used in this study to estimate person and item parameters for the CVA subscale include the *Simple/Dichotomous*, *Rating scale* and *Partial credit* as well as the *Group model* [12]. The probability of responses in the Rasch analysis is based on two facets: a person facet and an item facet. There are two key assertions that are assumed in the Rasch models used [12]. For the NBI scales the assertions are the following: (1) The more neurobehaviorally disabled a person is, the more occupational errors impacting performance will be scored as present; (2) Errors that emerge with a mild NB impact are more likely to be scored as present for all persons than those that only emerge with severe NBIs [6,15].

The original NBI CVA subscale of the A-ONE was based on the simple or dichotomous Rasch model, including items from both of the NB subscales of the instrument [6,15]. This is because the *Rasch Rating scale model* used in the analysis of the ADL scale requires that all items share the same rating scale and, thus, have the same number of categories. Therefore, to keep the needed dichotomous NBPIS items on the Rasch-based CVA scale, all 5-level rating scale items on the NBSIS were dichotomized (present/absent) by collapsing the categories [6].

Prior to analysis of the data with the dichotomous model in the initial Rasch analyses of the NBI scale [6], a content analysis of the items had been performed on the two subscales to examine their potential for inclusion in the study. The results of the content analysis of the A-ONE NB scale led to the inclusion of 37 NBSIS subscale items and 18 NBPIS subscale items for a total of 55 dichotomous items, 53 of which were retained after the updated Rasch analyses of the dichotomous CVA scale (refer to [6,15] for more details).

**Figure 1:**
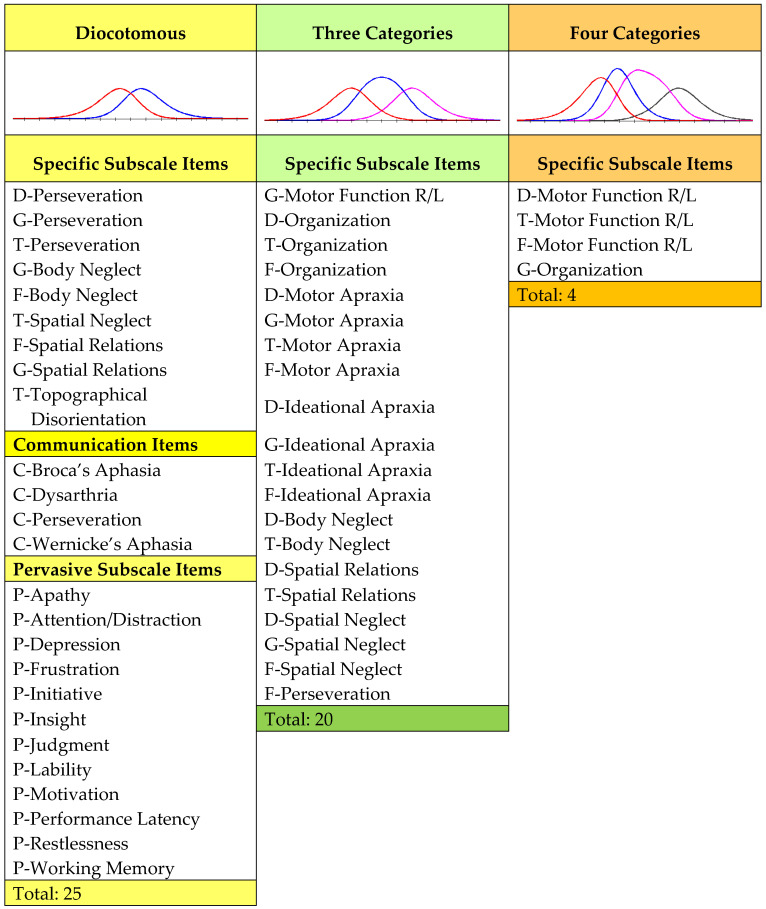
Item groups of the A-ONE CVA subscale. The items from the NBSIS are marked with capital letters referring to the five different domains. These are D = Dressing, G = Grooming and hygiene, T = Transfers and mobility, F = Feeding, and C = Communication. P refers to items from the NBPIP that remain dichotomous.

As the dichotomous scale has a restricted score range, it was of interest to see whether the score range could be increased by application of a different Rasch model for the software version of the CVA scale. Thus, before incorporating a CVA scale into the A-ONE software, we wanted to explore whether its rating scale range could be increased for some of the NB specific items by increasing the number of categories of the previously collapsed categories. Although our intention was not to use a separate rating scale for each item as is done when the *Partial credit Rasch model* is used, we decided to perform a Rasch analysis with it to explore which items had the potential to have the number of categories increased. We subsequently grouped the items based on the psychometrically sound number of rating scale categories into three groups of items, including items with two, three and four working categories. The criteria for analysis of the rating scale included, as in an earlier Rasch study of the A-ONE, a minimum of ten observations in each category, average category measures advancing monotonically with the scoring categories of the rating scale, and an outfit Mean Square (*MnSq*) value within 1.5; in addition, all thresholds should be ordered and advancement of calibrations between categories should be at least 1.4 logits [12,14,50]. Figure 1 demonstrates the classification of items into groups based on the number of working categories. Then we performed a Rasch analysis using the *Group model*, with the items grouped according to the number of active categories on the scale, and compared those psychometric scale results to the results of the previous CVA scale study with the *Dichotomous Rasch model*. The WINSTEPS Rasch computer software program version 4.7.0 was used in the studies [51].

To explore the internal validity and reliability of the A-ONE CVA subscale, we used a Rasch analysis based on scores from 222 individuals. Unidimensionality can be evaluated by the use of Rasch-based goodness-of-fit statistics to indicate how well the data fit the Rasch model assumptions and by principal components analysis (PCA) of the residuals [12,52]. Goodness-of-fit is determined by exploring the deviations of each item’s residual responses from the expectations of the Rasch model used. Two alternative statistics indicate the degree of fit of an item to the modeled underlying construct. These are the standardized (*z*) statistic and *MnSq*. The criteria we used both for the infit and outfit was based on a combined consideration of *MnSq* ≤ 1.4 and *z* < 2 [12,14]. If items did not meet these criteria, we planned to remove them one at a time, provided that the misfit exceeded the 5% of items that could be expected to misfit by chance [53]. We also considered item polarity, a dimensionality diagnostic revealed by the point-biserial measure [12]. Items with low correlation (<1.2) would be considered inconsistent with the impact scale measure and thus removed.

Reliability estimates are presented in the form of a standard error (*SE*) in Rasch analyses, both for each person and each item. The *SE* for persons is important for determining the sensitivity of the measures when evaluating changes. Reliability information is also revealed by the reliability coefficient (R), which reflects the replicability of person or item placements along the scale, and the separation index (G), which indicates the spread or separation in *SE* units. The separation index for persons indicates how well the items separate the entire sample of people into statistically distinct levels of ability [12].

#### 2.3.2. A-ONE Software Construction

**Figure 2:**
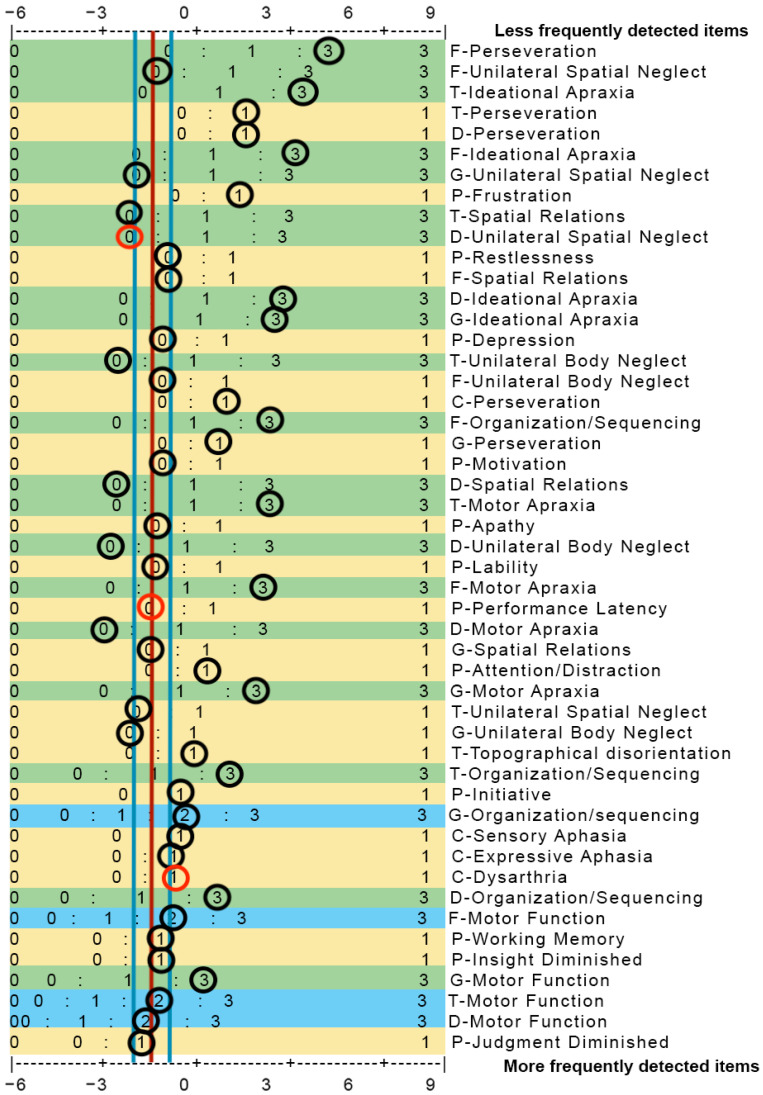
Key form for the CVA subscale and item hierarchy of the 49-item CVA scale. More frequently impacting items are listed at the bottom of the hierarchy on the right side of the figure. The rating scale for each of the tree groups of working categories range from 0 (no impact) to 3 (physical assistance needed to overcome the item’s impact). “:” represents the threshold between rating categories (50/50% chance of either score). Lower-impact ratings are located on the left side and more impacting ratings, reflecting a need for more assistance, on the right side. Background shades demonstrate the number of category ratings, based on the grouping for each of the items (yellow = dichotomous; green = three categories; blue = four categories). Circles indicate a rating sample for a patient diagnosed with LCVA. Black circles indicate detected ratings; red circles represent predicted missing scores. The red vertical line represents the person’s measure, with the blue lines on either side indicating the *SE*.

The main goal of the software project was to design efficient and time-saving software that is easy to use in the clinic. The computational model constructed for the CVA scale applies the Rasch-based measurement values developed by the *Group model* for score conversion. The computational model includes (a) the development of an algorithm for missing data imputation and (b) a calculation of statistical significance when comparing different measures. The algorithm was based on a probability table (score profile also referred to as key form) representing the estimated score distributions for ordinal scales with two, three and four category levels, as well as adjusted for the difficulty levels of 49 impairments that impact the performance of the 20 ADL tasks (see Figure 2 for the key form developed). The algorithms use the conversion and probability tables to map scores to logit measures. The expected scoring forms a vertical line that minimizes the total sum of distances to the data points in a one-dimensional optimization problem, useful for correcting for sampling bias. The constructed computational model and the statistical methods called upon for the report generation of incomplete data and to correct for sampling bias were all implemented in Python version 3.10. The imputation logic handles up to four missing score values. The software was set up using the web framework Django, backed by a relational database. Appropriate Django security features were used for authentication and authorization within the software.

#### 2.3.3. External Validity of the A-ONE CVA Software Subscale

We then turned to examine the external validity of the updated CVA scale by comparing its software-based measures and *SE*s to the measures obtained from the Winsteps-based Rasch analysis of the data. The criteria for difference were set at ≥0.5 logits. We also planned to explore the significance of obtained differences with a two-tailed paired t-test using Microsoft Excel 2021.

#### 2.3.4. Examination of the Intervention Program

The occupational therapy program at the rehabilitation unit of Landspítali is based on a three-phase service matrix developed in 2014. The construction of the matrix included assessment, intervention and re-assessment phases of the *Occupational Therapy Intervention Process Model (OTIPM)* [27,54,55] but was extended to include the classification of assessment methods, type of assessment, as well as categories and examples of intervention methods. The six activity groups for intervention in the OTIPM at the time of the service matrix development included the three occupation-based categories (*Restorative occupation*, *Acquisitional occupation*, *Adaptive occupation*) as well as three other categories (*Preparation*, *Rote practice/exercise*, *Simulated occupation*). These categories have been described earlier in more detail [27,54,55]. Later, the seventh intervention group was added to the OTIPM, and it was classified as *Occupation-focused educational programs for groups* [27]. Several studies [55] have previously been performed at the rehabilitation unit of Landspítali by applying the information in the intervention step of the service matrix using the six activity groups described by Fisher to examine whether the intervention methods used could be considered occupation-based.

**Figure 3:**
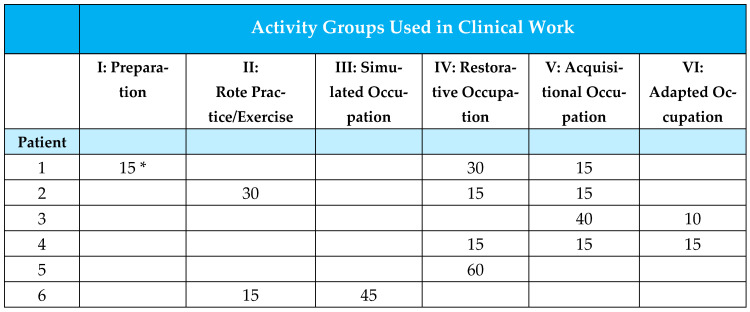
Time record for use by activity groups. * Time unit is minutes.

For the present study of CVA patients, four rehabilitation unit therapists recorded the time used in each of the six activity groups over one week. Specific guidelines were prepared. Figure 3 demonstrates the form used for recording the intervention time for each activity group. The percentage of total intervention time in minutes, used for each activity group, was calculated, as well as the percentage of time reflecting the use of occupation-based methods. As the later-developed *Occupation-focused educational program for groups* was not a part of the earlier version of the OTIPM and no classroom group sessions were scheduled for the week of the study, we chose to leave it out for the purpose of comparison to earlier study results.

The figures are based on therapists’ real documentation of the activity groups. Use of more than one activity group is possible during each therapy session.

The intervention part of the study was carried out to explore if intervention practices or emphases had changed over time. In accordance with the study conducted in 2016 [55], our occupational therapists registered the time spent with each patient over one week and classified it into the six available categories.

#### 2.3.5. Exploring the Significance of Outcome Measures Based on the A-ONE Software Scales

The inclusion criteria for the pilot study required that a patient had been diagnosed with CVA or stroke due to hemorrhage or ischemia, had obtained occupational therapy intervention as an inpatient at the rehabilitation unit and had been evaluated with the software version of the A-ONE both before and after the intervention. All patients received ADL intervention sessions four to five times a week in addition to daily intervention sessions at the occupational therapy department. Each session ranged from 30–60 min. Intervention time for patients in the outcome study ranged from 1–14 weeks (mean = 5.7 weeks; SD = 3.5). Pre- and post-intervention evaluation scores were entered into the A-ONE software to obtain information on the statistical significance of differences on both the ADL and CVA scales.

## 3. Results

### 3.1. Internal Validity

Prior to running Rasch analyses to explore unidimensionality and other psychometric properties of the CVA scale, we ran analyses of the psychometric properties of the rating scale categories based on the three groups. The results of the category measure analysis met all the set rating scale criteria for all three item groups. Three items demonstrated point-biserial measures < 0.12 (*Confabulation*, *Confusion*, *Irritability*) and were removed from the scale before the fit analysis. All remaining items met the set *MnSq* criteria of <1.4 (infit range = 0.48–1.28; outfit range 0.49–1.31). Other results from the construction of the Rasch-based A-ONE CVA subscale, including internal validation studies and reliability of measures, are summarized in Table 2. Results from the Rasch study with the previous dichotomous model are also shown for comparison. The number of categories in the *Group model* analyses exceeds the dichotomous categories in the *Simple Rasch model*. Person separation (>2) shows that the items divide persons into more than three performance layers and can thus be used to detect changes in performance. The score range of the Group model is higher than the score range of the *Simple model*, so the aim of increasing the score range by using the *Group model* as opposed to the *Dichotomous* one was achieved.

### 3.2. The A-ONE Software and External Validity of the CVA Subscale

**Figure 4:**
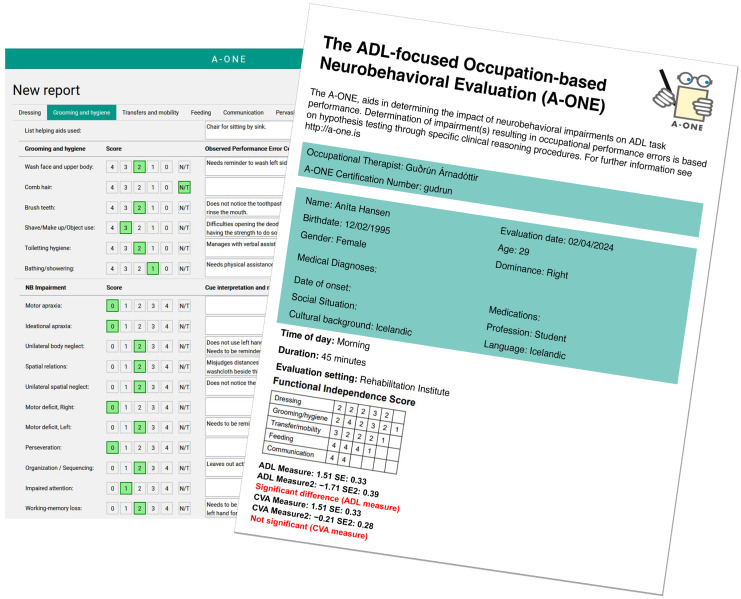
Software forms. The illustrated forms show the data entry form of the A-ONE software used to construct the evaluation reports and an example of a summary sheet from the digital report. Comparison of two different performance measures obtained at different times indicate statistically significant improvement in ADL performance but non-significant changes in the impact of impairments on the CVA scale in this particular report.

The score form from the digital report of the A-ONE software is illustrated in Figure 4. The figure also displays the comparison of two different performance measures obtained at different times, both for the ADL scale and the CVA scale.

To explore the external validity of the CVA subscale, person measures from the CVA software-based measures were compared to measures obtained from the Winsteps Rasch analysis program. A non-significant measurement difference (<0.3 logit) was detected between the A-ONE CVA scale software and Winsteps for each individual. Paired t-tests were also performed. The results indicated that the software computations were not significantly different from the Winsteps measures (*p* = 0.3). The two sets of measures were thus considered to be identical, with a logit range of differences of between 0.00 and 0.25, as shown in Table 3.

### 3.3. Intervention Study

The four occupational therapists recorded 65 stroke patients’ intervention sessions during the week out of their 129-session total workload. All inpatients at the rehabilitation unit received occupational therapy intervention five times a week in addition to daily ADL training. Sessions lasted 30–60 min. The results from exploring the nature of intervention administered to stroke patients at the rehabilitation unit of Landspítali revealed that 66.4% of therapists’ intervention time was devoted to the three occupation-based categories (*Restorative occupation* (IV = 46.5%), *Acquisitional occupation* (V = 16.9%), *Adaptive occupation* (VI = 2.9%), *Preparation* (I = 0.4%), *Rote practice/exercise* (II = 10.1%) and *Simulated occupation* (III = 23.2%); see Table 4 for more details on the differentiation of recordings between activity groups.

### 3.4. Outcome Study

The range of the A-ONE Rasch-based ADL software measures is from −7.48 logits (severe dependence) to +6.42 logits (independent). The pre-post-intervention comparisons between the first ADL evaluation (mean = 0.12; SD = 1.2; range = −2.02–2.88) and the second one (mean = 1.8; SD = 2.03; range = −2.02–6.42) provided by the software ADL scale revealed statistically significant results in 90.5% of the comparisons. The logit range of the A-ONE CVA scale measures range from −6.97, indicating no impact of neurobehavioral impairments, to +6.94, revealing a severe impact. The range of CVA scale comparisons between the first evaluation (mean = −1.82 logits; SD = 0.84; range = −0.7 to −3.72) and the second one (mean = −2.53; SD = 0.11; range = −0.7 to −0.5.07) revealed a statistically significant difference in 36.4% of the comparisons. A paired t-test revealed a statistically significant difference of means on both the ADL scale (*p* < 0.001) and the CVA scale (*p* < 0.001).

## 4. Discussion

The A-ONE instrument has developed through several cycles, including historical, educational and measurement cycles [8]. In this paper, we demonstrate how a new cycle of the A-ONE development was created by the construction of Rasch-based software from equal interval scales, including the CVA subscale. We have further illustrated how the CVA and the ADL scales can be utilized to measure statistically significant improvements, not only for clinical purposes but also for research. The NBI CVA software scale created is psychometrically sound in terms of its internal and external validity as well as reliability. The scale can be used to detect statistically significant changes in the impact of NB impairment on the ADL performance of CVA patients. The following topics will be used to cover the four research questions posed in the introduction: (1) construction of an internally valid Rasch-based A-ONE NBI CVA subscale, (2) incorporation of a renewed CVA scale into the A-ONE software, (3) content of the intervention program and (4) A-ONE software detection of improved ADL performance and the diminished impact of impairments on the performance. Finally, the fifth topic, summarizing the results, describes the addition of a new developmental cycle of the A-ONE.

### 4.1. Construction of an Internally Valid Rasch-Based A-ONE NBI CVA Subscale

Different neurological diagnoses produce different patterns of impairments that may affect task performance differently [5,6]. Prior to software construction, there were several decisions to be made regarding scale inclusion, as possible scales could be either generic or diagnosis-specific. There are pros and cons of both types of scales [6,15]. Item hierarchies of generic scales such as the All Diagnosis (All-Dia) NBI subscale of the A-ONE software can be used to compare individuals with different diagnoses, but they will not focus specifically on impairment groups related to a particular diagnosis. In contrast, item hierarchies of diagnosis-specific scales focus more on a certain pattern of impairments that results from a particular diagnosis such as CVA, but they are less suitable for the comparison of individuals with other diagnoses. Different diagnostic subscales thus have different item hierarchies. All the NBI items on the A-ONE are included in the software and enter the different diagnostic scales when called upon by the therapist’s choice of a particular subscale hierarchy. In each situation, items not included in the chosen hierarchy and the measurement model of the chosen scale remain in the A-ONE software pdf reports, including descriptive information and comments, but they do not contribute to the measurement-related values.

Clinical usefulness is another important consideration in scale construction. In the introduction of this paper, we brought up the issue of therapists reporting problems with time limitations when it comes to the use of standardized assessments and the conversion of rating scale values into measures [2,18,21]. Although scales for more precise sub-diagnoses, such as RCVA and LCVA scales, could be developed [6,15], incorporation of two scales based on different item hierarchies would limit possibilities for the comparison of measures from individuals within the two groups. An increased number of subscales would also require increased time for decision making regarding which subscale to use for which patients and when. We, therefore, chose to include a combined CVA scale in the A-ONE software to start with, in addition to the global NBI scale intended for all neurological diagnoses. We used the previously developed CVA subscale [6,44] as an item template, but we chose to explore if the score range could be expanded to make better use of the obtained information, as polytomous items always provide more information than dichotomous ones [12].

We were able to apply a different Rasch model from the previously used *Dichotomous model* by constructing three item groups. This was obtained by first applying the *Partial credit Rasch model* [12] to look at a number of active item categories on the rating scales for each item. Subsequently the data was analyzed using the *Group model* [12] with two, three and four active categories. This resulted in a 49-item internally valid and reliable CVA scale according to the criteria used. By this method, we managed to increase the scoring categories from two to nine and the score range of items from 53 to 78. Additionally, the *MSE* decreased slightly from 0.47 to 0.40. Other values were more or less identical on the original and renewed CVA scales.

Comparing the item hierarchies of the renewed CVA scale, based on the *Group model*, to the original dichotomous CVA hierarchy [6] revealed that the location of the four *Motor* impairment items was very similar, with these items being located as the most frequently detected items on both hierarchies. Thus, although the *Motor* items on the renewed scale now consist of three to four categories each, instead of two earlier, their location on the hierarchy is unchanged. Regarding whether the CVA scale hierarchy seems to be trustworthy, having *Motor* impairment items, which occur both with right and left CVAs located on the item hierarchy as the most frequently detected items, is in agreement with the literature, where motor problems resulting from a CVA are reported to be among the most frequently detected impairments [11,19,58,59]. Less frequently detected items located at the other end of the two hierarchies also included similar items on the two versions of the scale, including *Ideational apraxia*. Having *Ideational apraxia*, an impairment associated with severe disability, at the less frequently detected end of the hierarchy, as compared with the more common and less severe *Organization and sequencing* items, located with *Motor items* at the opposite end of the hierarchy, is also convincing.

**Figure 5:**
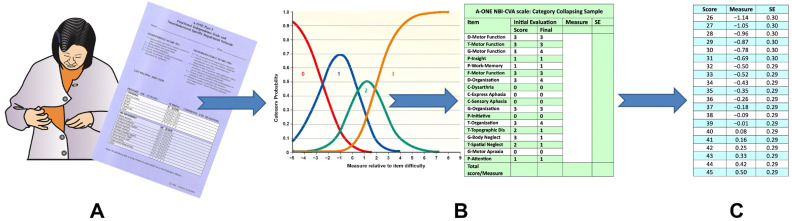
Manual conversion of rating scale scores into measures. (**A**) Documentation: performance of ADL tasks and detection of impacting impairments. (**B**) Manual combination of scoring categories: a combination of two categories on the ADL scale is required before conversion to measures, but two, three or four categories are required on the NBI scales depending on the item groups. (**C**) Sample from the A-ONE CVA scale conversion table. Reliable measures based on a score table require that no rating scale values are missing.

Before the A-ONE software was constructed, therapists using the A-ONE could obtain measures by using raw-score conversion tables, as mentioned in the introduction. Then, when comparing values from two evaluations before and after intervention, one of the measures would be subtracted from the other. Subsequently, the two *SEs* from both evaluations were summed and compared to the difference obtained from the pre- and post-measures. If the difference was greater than the sum of the *SEs,* then one could assume a statistically significant improvement [4]. Score conversion tables, however, cannot be used when ratings are missing, as occurs clinically at times. In such instances, one would need to turn to the key-form solution for estimating the missing scores. Figure 5 shows the needed steps in the process of converting rating scale scores into Rasch-based measures by the use of conversion tables.

As was pointed out in the introduction, there is a possibility of using the key maps to predict values for missing scores [16,17,18]. This could be useful for scales composed of five items such as the cognitive items of the FIM [16] and might even work for 20-item scales such as the ADL scale items of the A-ONE. However, when the number of items approaches 50, like on the CVA subscale, managing key maps is likely to be too time consuming and cumbersome for clinical use (refer to Figure 2 in the Methods section for an example of a filled-in 49-item key form from the CVA scale). Therefore, development of the A-ONE CVA scale software became crucial.

### 4.2. Incorporation of a Renewed CVA Scale into the A-ONE Software

As has already been pointed out, the number of items of the neurobehavioral subscales in the A-ONE software, including the CVA scale, varies with the item hierarchies, depending on which items are fit to be included on the particular scale. Additionally, the three item groups have different numbers of categories, resulting in conversion tables with various numbers of scores. This variability did complicate the work involved in constructing mathematical models for the different NBI subscales, including the CVA scale. Items are also placed on the hierarchy in different orders based on the item difficulty and the structure of the individual diagnostic subscales. The mathematical model developed takes items of different difficulty levels into consideration based on the type of scale as well as the different number of categories, including dichotomous, three- and four-step categories.

### 4.3. Content of the Intervention Program

Numerous studies and systematic reviews [58,59,60,61] have been performed in recent years to explore the efficacy of different types of intervention methods used in occupational therapy and rehabilitation. These include exploration of the effect of intervention methods used for patients with motor impairments [62] and cognitive impairments [63,64], as well as the effect of occupational therapy intervention methods on ADL and cognitive and physical functions [65]. Our aim in reporting on the content of the intervention program used in the pre–post outcome study of the A-ONE software was, on the contrary, to provide a description of the nature of the intervention program used, including the six activity groups, three of which relate to occupation-based methods, not to study the effectiveness of specific types of intervention. Thus, any direct comparison of the program’s content, which includes a combination of several methods with cognitive, perceptual and motor focus in addition to task performance, to results from effectiveness studies is beyond the scope of this paper.

The intervention program included two daily occupational therapy sessions on most days for all CVA inpatients at the rehabilitation unit. This is a considerable amount of intervention, as supported by results from the recent survey of hospital-based occupational therapists in Denmark working with CVA patients, where the 23% of therapists with the highest number of intervention sessions covered 5–7 sessions per week [2]. Our length of individual sessions of 30–60 min was comparable to findings from the Danish survey. In our study, 66.4% of the intervention time included occupation-based intervention from one of the three activity groups. The most frequently used group was *Restorative occupation* (46.5%), followed by *Acquisitional occupation* (16.9%) and then *Adaptive occupation*, which accounted for only 2.9%, as detected during our one-week study. This is the same sequence of activity group frequencies as obtained in the Danish survey of therapists, although the percentages there were based on therapists’ opinions, not documentation of content during intervention sessions. A one-week intervention study similar to ours at the same rehabilitation unit in Iceland was performed in 2016. Five occupational therapists treated 40 patients, including 21 CVA patients. The proportion of occupation-based intervention (67%) in the previous study was very similar to ours [55]. We are, however, aware that the suitability of activity types chosen at any given time depends on the time from insult, its severity and the patient’s condition, which create some variability [55]. A recent Canadian systematic review of the effectiveness of occupational therapy interventions on ADL performance, cognitive and physical function [65] reported a similar duration of intervention to that in our study, ranging from 2–12 weeks, with 3–10 sessions per week, each lasting 20–60 min.

### 4.4. A-ONE Software Detection of Improved ADL Performance and Diminished Impact of Impairments on Performance

Significant improvement in ADL task performance in the pilot study of 21 comparisons was achieved in 90.5% of the comparisons. The two 62-year-old male patients that did not demonstrate statistically significant measurement differences in their ADL performance had received intervention for four weeks each. Most of the recovery in ADL is reported to occur within the first six weeks after the incident [66]. Based on this information, many of the patients in our study may have received all the intervention they could make use of for ADL improvement in their program, which lasted 5.7 weeks on average.

Many of the ADL evaluations used in rehabilitation have ordinal scales, and some are based on questionnaires or interviews as opposed to observation, thus making direct comparison of research results difficult. Significant reduction (*p* < 0.001) in ADL dependence was, for example, reported in a recent Swedish study three months after the stroke occurred (*n* = 366) as compared with evaluation results from two days after the incident. However, the Barthel Index, which is a descriptive ordinal rating scale [67,68], was used for the initial evaluation, and then a self-reported questionnaire was used for the follow-up; this is in comparison with the equal-interval scale of the A-ONE software based on evaluation of observed performance.

The decrease in impact of impairments revealed by the CVA software scale was statistically significant in 36.4% of the comparisons. The intervention time for eight patients, aged between 55–76, who had a significant reduction in the impact of impairments on task performance ranged from 2–14 weeks. The A-ONE NBI CVA software scale is used to evaluate the impact of neurological impairments, be they of motor, cognitive, perceptual or emotional origin, on activity performance. The item content is, thus, different from traditional evaluations used to assess neurological impairments, where impairments of motor, cognitive, perceptual and emotional origin are evaluated separately and out of the natural context. Thus, any direct comparison of results will be difficult due to the nature of the items. In light of results from a follow-up study of change in seven different cognitive domains [69] of 153 stroke patients, where cognitive impairments were reduced by 8% in six months, one could speculate as to whether a longer duration of our intervention program (range = 2–14 weeks) would have detected further improvements in cognition.

Considering that the impact reduction on the A-ONE CVA software scale was considerably less than the obtained performance improvement on the ADL scale, it would also be interesting to explore if the difference could possibly be partially explained by the improvement in ADL performance being related to different types of activity groups in the intervention program, three of which are occupation-based and ADL-focused, whereas only one of the three, *Restorative occupation*, is additionally focused on impacting impairments. Thus, although impairments like paralysis may not decrease, people can learn to compensate for it by learning new methods (*Acquisitional occupation*) or by the use of aids (*Adapted occupation*).

### 4.5. New Cycle of A-ONE Development

**Figure 6:**
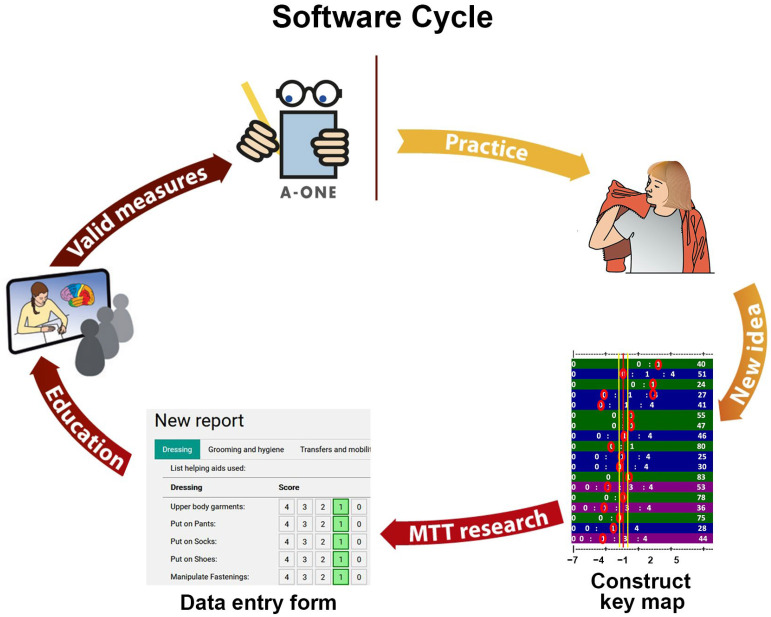
The software cycle of the A-ONE. The idea for the A-ONE software construction developed as result of clinical therapists being reluctant to use score tables for converting ordinal scale scores into measurements. This led to the construction of mathematical models based on Rasch key forms. The software is based on research on the internal and external scale validity for both the ADL and NBI scales. The software and the research studies behind it have been presented at international conferences. Benefits of the cycle that can flow back into clinical practice include access of A-ONE trained therapists to software measures and information on its use. (Adapted from Árnadóttir G. *Measuring the impact of body functions on occupational performance: Validation of the ADL-focused Occupation-based Neurobehavioral Evaluation (A-ONE)* p. 47. The Author, Reykjavík, Iceland, 2010 [6]).

The idea of software development for the A-ONE arose as a result of external factors, which include the requirement for the use of interval scale measures in rehabilitation services. Thus, despite the existence of evaluation tools with measurement qualities and an identified need for the use of available valid and reliable outcome measures [9], therapists have been reluctant to use these based on a reported lack of time and resources [18,19,20,21]. These dilemmas resulted in the successful development of software through the use of mathematical models based on Rasch-analyzed key forms of the A-ONE scale items and the potential for clinical use of the A-ONE scales as a linear measure. We succeeded in creating both internally and externally valid scales, including a CVA subscale, that could be used to detect statistically significant changes in the impact of NB impairment on ADL performance of patients diagnosed with CVA. The results of the validity and reliability studies have been presented at international conferences (including the most recent World Congress of Occupational Therapy in 2022 and the Frist Occupational Therapy Europe Congress in 2024), published in OT journals [24] and distributed in the form of printed lecture notes (see Figure 6 for an illustration of this process).

The sample size in the retrospective pre–post-test pilot study is small (21 comparisons), as it was limited to those CVA patients at the rehabilitation unit that had received a re-evaluation with the A-ONE. A bigger sample will be desirable in order to allow for generalization based on the results. A bigger sample is likely to be more representative of the CVA population, both in terms of impairment level and ADL performance. Nevertheless, this small sample can still provide meaningful insights into the usability of the A-ONE software. We limited our focus to a description of the occupational therapy intervention program, not intervention by other services. Although there was no control over how much impact other services may have had on the outcome, the study does cast some light on the content of the occupational therapy intervention provided. This paper is limited to the construction of one subscale, but other A-ONE NBI subscales could also be created and incorporated into the software if desired.

For future considerations, it would be desirable to perform a similar study with a larger sample size in terms of the pre–post-intervention pilot study of the A-ONE. In terms of the A-ONE software, studies exploring the possibility of diagnosis-specific scales for the ADL scale and an examination of whether Rasch models other than the *Rating scale model* would provide more useful information would be of interest. Further examination of the NBI scale items might provide an increased number of subscales for diagnostic groups other than CVA and the All-Dia scales that could be included in the A-ONE software.

## 5. Conclusions

We succeeded in reaching our goal to expand the A-ONE software by developing a Rasch-based CVA subscale that can detect statistically significant changes in the impact of neurobehavioral impairments on task performance. This is, to our knowledge, the first study to deliver a clinically deployable Rasch-based CVA subscale integrated into routine occupational therapy software. The software offers considerable time savings for occupational therapists who use the A-ONE either clinically or as a research tool. In addition to estimating missing values, converting rating scale values into measures and providing a computerized report, it simultaneously allows for the comparison of different measures and tests of statistical significance. The fact that it allows for documentation of ordinal A-ONE scores and their conversion into measures contributes to the removal of clinical obstacles toward the use of the A-ONE as an outcome measure and encourages the use of measures in evaluating outcomes in rehabilitation.

## Figures and Tables

**Table 1:** Age and gender of participants in the internal validity study by diagnostic group.

	CVA		Total
	Left	Right	
**Age**			
Mean	67.7	65.3	66.6
SD	13.5	14.3	13.9
Range	22–89	22–91	22–91
**Gender n (%)**			
Male	74 (55.6%)	59 (44.4%)	133 (59.9%)
Female	40 (44.9%)	49 (55.1%)	89 (40.1%)
**Total n (%)**	114 (51.4%)	108 (48.6%)	222 (100%)

Note: CVA = cerebral vascular accident.

**Table 2:** Psychometric properties of the A-ONE CVA scale obtained by two different Rasch models.

Psychometric Test Person/Item Information	Rasch Model		Criteria	Criteria References
	Simple	Group		
Number of persons	222	222	≥150	[12,49]
Item number	53	49	≤53	
Number of categories *	2	2, 3, 4 = 9	>2	
Item Infit Misfit	0	0	*MnSq* ≥ 1.4; *z* > 2	[12,14,56]
Item Outfit Misfit	1	0	*MnSq* ≥ 1.4; *z* > 2	[12,14,56]
PCA: First contrast	10%	10%	≤10%	[57]
Person separation	2.24	2.26	≥2	[12,14,56]
Person reliability coefficient	0.83	0.84	≥0.8	[57]
Item reliability coefficient	0.98	0.97	≥0.8	[57]
*MSE* persons *	0.47	0.40	≤0.5 logit	[12]
*MSE* items	0.24	0.20	≤0.5 logit	[12]
Score range *	0–53	0–77	>0–55	

* Differences in favor of the Group Model.

**Table 3:** Comparison of CVA software scale measures and Winsteps measures.

Measurement Difference of Winsteps and A-ONE Software								
Persons	Age	Gender	A-ONE Measure *	A-ONE SE	Winsteps Measure	Winsteps SE	Measure Difference	SE Difference
1-CVA-L	82	M	−0.79	0.29	−0.69	0.29	0.10	0.00
2-CVA-L	73	F	−2.97	0.40	−2.94	0.39	0.03	0.01
3-CVA-L	72	M	−2.27	0.35	−2.29	0.34	0.02	0.01
4-CVA-L	75	M	−2.53	0.37	−2.53	0.36	0.00	0.01
5-CVA-R	22	M	−3.50	0.45	−3.45	0.44	0.05	0.01
6-CVA-R	62	M	−2.40	0.36	−2.29	0.34	0.11	0.02
7-CVA-R	84	F	−3.31	0.43	−3.26	0.42	0.05	0.01
8-CVA-L	83	M	−1.32	0.31	−1.22	0.30	0.10	0.01
9-CVA-R	73	M	−1.51	0.31	−1.56	0.31	0.05	0.00
10-CVA-L	73	F	−1.61	0.32	−1.51	0.31	0.10	0.01
11-CVA-R	70	M	−3.72	0.48	−3.66	0.47	0.06	0.01
12-CVA-R	80	F	−0.53	0.29	−0.55	0.29	0.02	0.00
13-CVA-R	71	F	−3.96	0.51	−3.89	0.50	0.07	0.01
14-CVA-R	75	M	−2.04	0.34	−2.06	0.33	0.02	0.01
15-CVA-R	72	M	−2.15	0.34	−2.06	0.33	0.09	0.01
16-CVA-R	61	M	−4.04	0.75	−4.17	0.55	0.13	0.20
17-CVA-R	70	M	−3.13	0.41	−3.10	0.40	0.03	0.01
18-CVA-R	70	M	−2.81	0.39	−2.79	0.38	0.02	0.01
19-CVA-R	54	M	−1.82	0.33	−1.57	0.32	0.25	0.01
20-CVA-L	60	F	−2.53	0.37	−2.53	0.36	0.00	0.01
21-CVA-L	78	F	−1.51	0.31	−1.56	0.31	0.05	0.00
22-CVA-L	73	M	−1.61	0.32	−1.56	0.31	0.05	0.01

* A-ONE software allows for up to 4 missing values.

**Table 4:** Recordings of activity groups used during intervention sessions.

		Activity Groups Used for Intervention					
Therapist	Sessions	I	II	III	IV	V	VI
1	22	0 *	225	470	445	0	30
2	23	10	35	0	380	295	45
3	11	0	0	60	140	80	0
4	9	0	0	235	235	60	0
**Total**	65	10	260	600	1.200	435	75
**(%)**		0.4%	10.1%	23.2%	46.5%	16.9%	2.9%

* Time unit is minutes.

## Data Availability

The data that support the findings of this study will be made available from the corresponding author, G.Á., upon reasonable request. The data are not publicly available due to privacy and ethical clinical restrictions.

## Abbreviations

The following abbreviations are used in the manuscript:

ADL	Activities of daily living
ALL-DIA	All Diagnoses subscale
A-ONE	ADL-focused Occupation-based Neurobehavioral Evaluation
CTT	Classical Test Theory
CVA	Cerebrovascular Accident
FI	Functional Independence
FIM	Functional Independence Measure
G	Separation Index
LCVA	Left Cerebrovascular Accident
*MnSq*	Mean Square
*MSE*	Mean Standard Error
MTT	Modern Test Theory
NB	Neurobehavior
NBI	Neurobehavioral impact
NBPIS	Neurobehavioral Pervasive Impairment Subscale
NBSIS	Neurobehavioral Specific Impairment Subscale
PCA	Principal Components Analysis
R	Reliability Coefficient
RCVA	Right Cerebrovascular Accident
